# The development of an intervention to promote adherence to national guidelines for suspected viral encephalitis

**DOI:** 10.1186/s13012-015-0224-2

**Published:** 2015-03-20

**Authors:** Ruth Backman, Robbie Foy, Benedict Daniel Michael, Sylviane Defres, Rachel Kneen, Tom Solomon

**Affiliations:** Department of Clinical Infection, Microbiology and Immunology, Institute of Infection and Global Health, University of Liverpool, Ronald Ross Building, 8 West Derby Street, Liverpool, L69 7BE UK; Leeds Institute of Health Sciences, University of Leeds, Charles Thackrah Building, 101 Clarendon Road, Leeds, LS2 9LJ UK; The Walton Centre NHS Foundation Trust, Lower Lane, Liverpool, L9 7LJ Fazakerly UK; Royal Liverpool and Broadgreen University Hospitals Trust, Liverpool, L7 8XP UK; Department of Neurology, Alder Hey Children’s NHS Foundation Trust, Eaton Road, Liverpool, L12 2AP UK

**Keywords:** Theoretical domains framework, Intervention development, Encephalitis, Cluster randomised controlled trial

## Abstract

**Background:**

Central nervous system infections can have devastating clinical outcomes if not diagnosed and treated promptly. There is a documented gap between recommended and actual practice and a limited understanding of its causes. We identified and explored the reasons for this gap, focusing on points in the patient pathway most amenable to change and the development of a tailored intervention strategy to improve diagnosis and treatment.

**Methods:**

Using theoretically-informed semi-structured interviews, we explored barriers and enablers to diagnosing and managing patients with suspected encephalitis, specifically performing lumbar punctures and initiating antiviral therapy within 6 h. We purposively sampled hospitals and hospital staff in the UK. We audio recorded and transcribed all interviews prior to a framework analysis. We mapped identified barriers and enablers to the patient pathway. We matched behaviour change techniques targeting clinicians to the most salient barriers and enablers and embedded them within an intervention package.

**Results:**

We interviewed 43 staff in six hospitals. Clinical staff expressed uncertainty when and how to perform lumbar punctures and highlighted practical difficulties in undertaking them within busy clinical settings. Once treatment need was triggered, clinicians generally felt able to take appropriate therapeutic action, albeit within organisational and resource constraints. Matched behaviour change techniques largely targeted antecedents of treatment. These included decision support to prompt recognition, highlighting the consequences of missed diagnoses for clinicians and patients, and practical support for lumbar punctures. We subsequently devised an evidence-informed package comprising ‘core’ interventions and, to allow for local flexibility, ‘optional’ interventions.

**Conclusions:**

We identified several points in the patient pathway where practice could improve, the most critical being around clinical suspicion and initial investigation. Interventions targeting professional beliefs and behaviours whilst optimising their clinical environment were amongst the most promising approaches to improve the care of suspected encephalitis.

**Trial registration:**

Randomised trial registered with Controlled Trials ISRCTN06886935.

**Electronic supplementary material:**

The online version of this article (doi:10.1186/s13012-015-0224-2) contains supplementary material, which is available to authorized users.

## Background

There is accumulated evidence that the clinical management of a serious acute neurological infection does not meet recommended standards [1,2], resulting in lost years of life and quality of life [3]. Encephalitis is inflammation of the brain; it has a variety of causes [4], the most common being viral infection [5]. Encephalitis affects approximately five to eight people per 100,000 annually [6], with herpes simplex virus (HSV) encephalitis being the most commonly diagnosed sporadic viral cause [4].

National guidance in the UK currently recommends timely investigation and treatment for patients with HSV encephalitis [4,7]. Furthermore, treatment within a shorter time period is associated with improved patient outcomes [8,9]. Long-term outcomes from encephalitis are still not fully understood but sequelae include long-term disabilities such as concentration difficulties, behavioural and speech disorders, memory loss and epilepsy [3,5,10].

Encephalitis is challenging to diagnose, especially given the frequency of non-specific clinical presentations, its low incidence, and the subsequent risk of misdiagnosis in acute clinical settings [11,12]. Concern has also been expressed about the abilities of medical staff to perform the main diagnostic procedure, lumbar puncture [13].

Whilst a number of opinions have been expressed about why care is suboptimal [1,2,14,15], no research has sought to explain this gap between evidence and practice. Ideally, interventions to improve professional practice should be based upon a diagnosis of barriers to change, preferably focusing on those most amenable to change. The UK Medical Research Council framework for the development and evaluation of complex interventions advocates a systematic approach to intervention development, thereby potentially enhancing the targeting of active components of interventions [16,17]. We set out to develop a strategy to improve the diagnosis and management of suspected encephalitis by exploring barriers to, and enablers of, adherence to current guidance, matching these to behaviour change techniques which could be embedded within an intervention package.

## Methods

### Design

We undertook a qualitative interview study to explore health care professionals’ experience of, and beliefs about, diagnosing and managing suspected encephalitis (Table 1).

**Table 1:** **An overview of the intervention development process**

**Step**	**Sources and methods**
Identification of target clinical behaviours	Clinical guidelines; previous chart audits; discussion with clinical specialists
Exploration of barriers and enablers	Theory-informed interviews with hospital staff; discussion with clinical specialists
Identification of feasible intervention delivery methods	Systematic reviews; team discussion
Matching barriers and enablers to behaviour change techniques	Interview findings; taxonomy of behaviour change techniques; consensus
Embedding behavior change techniques within intervention delivery methods	Team discussion
Refining intervention	Piloting intervention components with targeted types of staff

There is a growing body of research on the beliefs and behaviours of health care professionals across a wide range of contexts [18]. Given its specific focus on potentially modifiable factors affecting clinical behaviour, we drew upon the Theoretical Domains Framework developed by Michie et al. [19] and later refined by Cane et al. [20]. The domains covered within the framework are constructed from 33 theories which can explain health care professional behaviour. These 12 domains comprise of: knowledge; skills; professional role and identity; beliefs about capabilities; beliefs about consequences; motivation and goals; memory, attention and decision processes; environmental context and resources; social influences; emotion; action plans and nature of the behaviour [19,20]. The framework has been applied across a range of settings and targeted clinical behaviours [21-26]. Whilst the Theoretical Domains Framework largely focuses on individual behaviours, we remained sensitive to wider organisational factors that influence the patient pathway by gathering field notes from observations and considering additional themes that might not fit within the framework [27].

### Participants and sampling

Our sampling frame consisted of hospitals within the Merseyside, Yorkshire and West Midlands regions in the UK. We recruited health care professionals (both physicians and nurses) from two specialist and four teaching hospitals using a diversity sampling approach to minimise geographical bias and identify a wide range of perspectives. To ensure a mix of staff grades, reflecting levels of training, within the hospital, recruitment was conducted using a purposive snowballing technique. We also opportunistically recruited consultants (fully trained physicians) from other hospitals who were attending an educational meeting to ensure some diversity of staff grades and levels of training, in the sample. We anticipated likely data saturation at between 30 and 40 interviews.

### Data collection

We developed a semi-structured interview schedule to elicit the barriers around two key clinical behaviours in the management of patients with suspected encephalitis: performing a lumbar puncture (the main diagnostic method for encephalitis) and recognising the clinical features of suspected encephalitis. We selected these two behaviours because they represented critical steps in the patient pathway which could also be measured as end-points for a subsequent randomised evaluation of a strategy to improve clinical management. Furthermore, we also examined the antecedents to lumbar puncture during the construction of the patient pathway. The schedule started with open questions and moved on to a series of prompts based upon the Theoretical Domains Framework (Additional file 1). We did this to avoid restricting participants’ responses and elicit issues that the framework might have missed.

We initially pre-tested the conduct and components of the interviews with three informal focus groups of professionals. Our observations of these led us to suspect that social desirability bias could influence responses, especially in group situations where respondents would feel less inclined to acknowledge their own uncertainties and shortcomings in the management of suspected encephalitis. We concluded that one-to-one interviews allowed individuals to reflect on their practice within a safe and less judgmental environment. We assured that interviews were not tests of knowledge and acknowledged variations in the current management of patients with suspected encephalitis.

All interviewees gave written consent. A health services researcher (RB) conducted all interviews. All interviews were audio-recorded and transcribed verbatim. We conducted interviews until we were confident that no new experiences or beliefs were being elicited, either within a specific staff training level or geographical location. We supplemented the interviews with a reflective log following each interview and field notes from contextual observations such as witnessing a lumbar puncture and observing teaching sessions for junior doctors at two different hospitals involving practicing of the procedure on a mannequin. Taking field notes informed our construction of the patient pathway and further guided both interview content and interpretation. These field notes were personal responses to what had been learned during interviews or observed during visits.

### Data analysis

The first ten transcripts were double coded (by RB and RF, a clinician and implementation researcher) using the Theoretical Domains Framework [20] to ensure consistency for barrier and enabler domain allocation. RB coded the remaining 33 transcripts and discussed any queries with RF. We constructed a prototypical patient pathway from interviews and field observations (Table 2).

**Table 2:** **Patient pathway with mapped barriers and domains**

**Patient pathway**	**Barrier identified**	**Domain**
Patient presents at hospital	Potential long wait time in Accident and Emergency	Environmental context and resources
Patient shows clinical features that indicate a lumbar puncture is required	Relies on clinical feature recognition	Knowledge
		Memory
		Beliefs about consequences
Decision is made to perform a lumbar puncture	May require several people to make this decision	Professional role
		Emotion
		Social influences
Patient is admitted to ward/medical admissions unit for a lumbar puncture to be performed	Patient will need to be re-clerked	Beliefs about consequences
		Environmental resources and context
Staff allocated to perform a lumbar puncture	Shift pattern, other duties, perceived importance of a lumbar puncture	Beliefs about capabilities
		Beliefs about consequences
		Emotion
Equipment and supervision found: Lumbar puncture ready to go ahead	Need to know what equipment is needed, where it is and finding someone to supervise	Knowledge
		Memory
Lumbar puncture performed	Many external factors as well as skill	Skills
		Beliefs about capabilities
		Emotion
Results available. Management plan made	Who can make the plan, delays with results and knowledge to interpret	Professional role
		Beliefs about consequences
		Social influences
Additional test needed	Who can action this? Further delays can occur here	Beliefs about social comparison
		Consequences
		Knowledge

### Intervention development

We aimed to develop a package of intervention components which could be flexibly and sustainably implemented within hospitals. RB initially matched domain findings to a set of defined behaviour change techniques [28], whilst actively considering what could be feasibly operationalised within our likely intervention resources and our own team abilities. A second researcher (RF) then checked the matched domains and behaviour change techniques to highlight possible gaps or redundancies. Overall, we went through four iterations of this process. We convened a sub-group of investigators, possessing specialist clinical knowledge in adult and paediatric neurology and infectious diseases. Drawing upon systematic reviews of the effectiveness of interventions to improve clinical practice [29-33], we then worked through how to incorporate the matched behaviour change techniques into an intervention package. We piloted and refined key components of the intervention prior to wider roll-out within a planned randomised trial.

### Ethics

Ethical approval for the interviews was gained (REC 11/EM/0442) as part of the ENCEPH UK Prospective study. Ethical approval for the ENECPH UK Intervention Cluster Randomised Controlled Trial was gained (REC13/NW/0279) prior to ISRCTN registration (ISRCTN06886935).

## Results

Forty-three professionals from two specialist hospitals and four teaching hospitals participated in interviews, comprising three consultants, 28 junior (training) doctors and seven nurses and health care assistants. Five consultants from other hospitals participated in the focus group discussion.

We constructed a simplified patient pathway as described in Table 2. We subsequently identified junctures within patient pathways that were susceptible to delays or diversions. The key problems in dealing with suspected encephalitis concerned initial clinical assessment and investigation. The initial assessment of suspected cases was influenced by the clinical environment and the limitations of professionals’ awareness and decision-making processes. Professionals, particularly less experienced medical staff, often lacked confidence in their abilities to perform lumbar punctures and encountered difficulties in arranging these within busy clinical contexts.

### Initial clinical suspicion and assessment

Knowledge, memory, attention and decision-making and environmental resources and context all appeared to have the greatest impact on initial clinical assessment. Recognising the non-specific clinical features of suspected encephalitis was the biggest challenge for doctors, regardless of training level.

*A 42 year old who came in with a first fit and no other symptoms at all, and then had a full fit. She had a CT [computed tomography] scan in A&E [accident and emergency] who couldn’t find anything wrong with her, referred her to the neurology registrar who saw her in the department and kind of wasn’t really sure what was going on. She went under the medics and was under the medics for two or three days before she sort of had any other symptoms, then started spiking a temperature and somebody sort of twigged.*

[Junior doctor [C], teaching hospital [2]]

The same doctor later reflected on how direct experience of encephalitis could influence thresholds for considering encephalitis in the future.

*Particularly with the first case I saw, well you just sort of knew she wasn’t right… and it took 48 hours before anyone thought of it [encephalitis]. We have now got, well I have got, a much higher level of suspicion if they have got neurological symptoms*.

[Junior doctor [C], teaching hospital [2]]

The busy environment and competing pressures within emergency departments constrain time for reflection and decision-making. There are constant pressures to keep patients moving through coupled with uncertainties about responsibilities and roles.

*We don’t have an A&E [accident and emergency] and that is the ongoing debate is how do we deal with acute neurology, it doesn’t apply just for encephalitis, it is for all acute neurological problems.*

[Consultant [D], specialist hospital [1]]

Furthermore, emergency department professionals rarely encounter or see the long-term influences of encephalitis and therefore may not prioritise rapid assessment. When weighing up perceived risks, short-term considerations took priority over longer-term consequences - even despite specialist advice that a lumbar puncture was indicated first in the following case:

*[The patient] came in. She had a few kind of slightly worrying signs on examination, in terms of focal neurology. So I spoke to the on-call radiologist who declined to do a CT [computed tomography] because he said there weren’t contraindications for just doing the lumbar puncture. I handed her over to the night time registrar when I left for the LP [lumbar puncture] to be done and when I came in the next day to check the LP hadn’t actually been done overnight because the registrar was worried about the focal neurology and would have preferred for a CT scan to have been done first. So in the end she was started on aciclovir and had a lumbar puncture after that and then a CT scan after that and she had confirmed encephalitis.*

[Junior doctor [C], teaching hospital [2]]

However, even if professionals recognised limitations in their knowledge, they found it difficult to locate point of care support for decision-making.

*I don't know whether the encephalitis guidelines are available, like I don't know if they are on the intranet here*. [Junior doctor [C], specialist hospital [1]]

*Down on A&E [accident and emergency] we have baskets with the actual paper copies [of pathways]… everyone knew there would be like a cellulitis pathway so if someone came in with cellulitis you would go and pick up the paperwork.* [Nurse [B], teaching hospital [2]]

### Investigation

Performing lumbar punctures competently and quickly whilst remembering to order all appropriate investigations were challenging for professionals within busy clinical contexts. They sometimes felt ill-prepared for all operational aspects of the procedure, from allocation of willing and competent staff to finding all the equipment and space to perform the procedure.

*Getting the sample pots that is the one thing I really remember from that one is just calling everyone absolutely everything, you have to go to microbiology, no its haematology, no its biochemistry, no it’s A&E [accident and emergency], and it was just a nightmare trying to get it and no ward seemed to have them and I think I finally got hold of one of them at biochemistry I think it was, and just the four different pots and things so it’s tricky.*

[Junior doctor [C], teaching hospital [2]]

Lumbar punctures require a level of acquired skill and are still perceived as being more risky and invasive than other procedures, given the location of the needle and the associated risks of brain herniation.

*Positioning, positioning the patient, everyone seems to be very keen about positioning. And erm increasing the space, all the things you can do to make it safe, that is all I have heard really.* [Junior doctor [C], teaching hospital [2], ahead of first lumbar puncture]

Furthermore, regardless of the level of clinical training, the participants expressed uncertainties around the volumes of cerebrospinal fluid (CSF) that can be safely collected. We observed the ENCEPH UK Chief Investigator (TS) dispelling a myth about risks involved in lumbar punctures by reassuring an educational meeting of consultants that ‘CSF [cerebrospinal fluid] is as precious a commodity as urine due to the same production rate’ (field notes).

Junior doctors found performing lumbar punctures stressful, especially when they had not done them before. However, they consistently reported that after performing their first successful lumbar puncture they were then able to perform them all successfully and also gained a sense of satisfaction.

*So, I actually did two successful ones [lumbar punctures] on the same day.*

[Junior doctor [C], teaching hospital [2]]

Field notes taken during interviews and focus groups suggested a gap between junior doctors’ confidence and their actual skill levels. During one focus group, all juniors reported feeling confident in performing lumbar punctures; they all subsequently struggled when practicing on a mannequin during the next session with most requiring senior level guidance and support.

We encountered uncertainty about roles and expectations, as the clinical context often generates ambiguous situations, in this case around paediatric care:

*There wasn’t anybody else who was able to measure or know how the technique of measuring the opening pressure. It was an older patient of 13 and I think that the paediatric trainees are used to younger babies and younger children as opposed to older children. I think the remaining staff who were with me felt they wanted me to undertake the procedure and I think one of the them stayed in the room to observe me doing the procedure as well.*

[Junior doctor [C], specialist hospital [1]]

### Initiating treatment

The participants indicated that the decision to initiate treatment with aciclovir was relatively straightforward once clinical suspicions had been triggered and a provisional diagnosis made. More concern was expressed about possible over-treatment and defensive medical practice.

*Why are we overly aggressive [at my place of work]? Well I think firstly the risk landscape is changing. I think different people are up to speed with that to a different extent and I think people’s perception of the risk landscape is an individual thing, it reflects their experience and the experience of their unit, but we are becoming more risk averse, the population is becoming less tolerant of rare events, if they have any predictability, and so I think in general we are moving to investigating more and treating more and trusting our clinical judgment less.*

[Consultant [D], teaching hospital [2]]

*If you remotely think it’s encephalitis in adults the patient gets bunged on aciclovir, the problem is the reverse; it is undoing the diagnosis.*

[Consultant [D], teaching hospital [2]]

There was an acknowledged risk that initiating aciclovir might then reduce motivation to undertake definitive investigation to establish the diagnosis.

*With suspected meningitis or encephalitis the patients get started on antibiotics and aciclovir and after that nobody really worries about doing the LP [lumbar puncture]. It can be two, three, four days after before the lumbar puncture is actually done.*

[Junior doctor [C], teaching hospital [2]]

Participants expressed further concerns around stopping treatment, especially in the absence of a final diagnosis.

*The aciclovir we had stopped after a week I think which is quite a short duration, when we didn’t really have an alternative diagnosis.*

[Junior doctor [C], specialist hospital [1]]

### Development of the intervention package

We initially matched 88 out of the available 93 behaviour change techniques to the 14 theoretical domains (Additional file 2). We then matched the intervention components to behaviour change techniques so that 43 were incorporated at least once within the intervention package, illustrated next with two examples.

Initial clinical assessment and recognition of suspected encephalitis depend upon an ability to respond and process information rapidly within a demanding clinical environment. Relevant behaviour change techniques include prompts for professionals to respond to salient clinical cues (thereby ‘conserving mental resources’) and reminders of the importance of timely diagnosis (reinforcing ‘salience of consequences’). Clinical guidance also needs to be accessible and embedded within existing point-of-care resources for decision support, recognising that these might be in paper or computerized formats across different hospitals.

There was a different and more variable set of challenges around performing lumbar punctures. Clinicians need support when deciding whether a lumbar puncture is indicated and safe to perform, which can come from one or both of point-of-care guidance or colleagues. Those who have not previously performed lumbar punctures require instructions and modelling, potentially coupled with a graded task approach whereby they practice and undertake lumbar punctures in progressively more challenging situations until they achieve a level of mastery. The graded task approach can also help to counter negative emotions and anxieties by developing confidence and allowing positive feedback on successes. The actual conduct of the lumbar puncture, particularly the need to assemble to correct range of tests, can be supported by ensuring ready access to pre-prepared equipment and instructions on which tests to request and how to take them.

We embedded these and other behaviour change techniques into an intervention package (Table 3). We applied the Template for Intervention Description and Replication (TIDieR) reporting checklist [34] and present this in Additional file 3. We defined ‘core’ interventions which we anticipated all hospitals being able to use, including educational meetings, newsletters with personalised audit data and provision of a lumbar puncture kit within a refillable box. To allow for local flexibility, we further defined ‘optional’ interventions which hospitals could use depending upon local resources and skills. These included decision support prompts such as phone apps and algorithms; an online quiz; personalised invitation letters to the educational meetings and a short audit pack with the basis of a modifiable care pathway. We presented the package to a 1-day meeting of senior doctors and nurses from intervention hospitals. We emphasised their roles in directly delivering the various intervention components locally and recommended that they each convene an action planning meeting on return to their hospitals.

**Table 3:** **Behaviour change techniques in the intervention**

**Intervention component**	**Selected behavior change techniques (definitions are available from Michie et al. 2013** **[** 28 **]** **)**
*Training day (core)*	• Identification of self as a role model
Investigators were invited to attend a training day where the intervention was showcased and key behaviour change techniques to be communicated to their trainees were covered.	• Instruction on how to perform the behaviour
	• Salience of consequences
	• Action planning
	• Credible source
	• Identity associated with changed behaviour
*Action planning meeting (core)*	• Action planning
After attending the training day, investigators were asked meet to plan how best to implement the intervention package. This included actively scheduling the educational sessions and assigning personnel to keep the lumbar puncture boxes refilled.	• Goal setting
	• Identification of selves as role models
	• Problem solving
	• Social support
	• Commitment
	• Review behaviour/outcome goal
*Audit and feedback newsletter (core)*	• Goal setting
A newsletter will be produced for local dissemination with personalised audit data which will be fed back to each hospital alongside a short clinical update.	• Discrepancy between current behaviour
	• Information about others’ approval
	• Salience of consequences
	• Anticipated regret and goal
	• Feedback on behaviour
*Lumbar puncture box (core)*	• Adding objects to the environment
A refillable box with all the key equipment to perform a lumbar puncture was provided with a page detailing sample collection [51] which could be locally modified as required.	• Instruction on how to perform the behaviour
	• Conserving mental resources
	• Restructuring the physical environment
	• Habit formation
	• Prompts/cues
	• Action planning
*Education (core)*	• Persuasive communication
Pre-made lectures with the behaviour change techniques were produced for the following uses: A session aimed at foundation doctors on how to perform a lumbar puncture; A session for the entire department focused upon the management of suspected encephalitis; and a session for nurses on how to help with lumbar punctures. These materials can be locally modified with a core set of slides so preserve behaviour change integrity. Furthermore, these are all modified for use in both an adult and paediatric setting and can be used as often as required by the local team.	• Instruction on how to perform the behaviour
	• Credible source
	• Information about others’ approval
	• Salience of consequences
	• Demonstration of the behaviour
	• Behavioural practice/ rehearsal
	• Discrepancy between current behaviour and goals
	• Anticipated regret
	• Feedback on behaviour
	• Action planning
	• Problem solving
	• Information about antecedents
	• Social support
	• Social reward
	• Commitment
	• Graded tasks
	• Review behaviour/outcome goal
	• Restructure the social environment
*Quiz (optional)*	• Action planning
An online multiple choice quiz was developed with tailored questions for doctors and nurses. This quiz can be used during educational sessions or within private study and all participants can download a certificate of completion.	• (Mental) behavioural practice/ rehearsal
	• Credible source
	• Discrepancy between current behaviour and goal
	• Information about consequences
	• Instruction on how to perform the behaviour
*ClickClinica (optional)*	• Conserving mental resources
An app was developed [52] as a tool for clinicians to quickly access up to date guidelines for all conditions. This has been promoted within our package both within the education and also within the personalized invitation letter as a useful tool.	• Instruction on how to perform the behaviour
	• Credible source
	• Behaviour substitution
	• Habit formation
	• Goal setting
	• Information about antecedents and outcomes
	• Personalised message
*Encephalitis Society leaflets and video (optional)*	• Social reward
The Encephalitis Society YouTube channel was included as a resource which could be incorporated into the education. Furthermore, patient leaflets will be disseminated to the investigators during the study.	• Credible source
	• Habit formation
	• Salience of consequences
*Short audit (optional)*	• Discrepancy between current behaviour and goal
A short audit featuring quality improvement cycles (plan, do, study, act PDSA) was developed and included a summary page with the key guideline recommendations along with a short list of key check box items to monitor current practice. An excel sheet which pre-plots the progress was included within the pack.	• Review behaviour goals
	• Social comparison
	• Anticipated regret
	• Feedback on outcome of behaviour
	• Self-monitoring of behaviour and outcomes
*Basis of modifiable care pathway (optional)*	• Conserving mental resources
The front sheet from the audit pack could also be modified to form the basis of a care pathway for suspected encephalitis patients. This will be locally driven and implemented at each site.	• Instruction on how to perform the behaviour
	• Habit formation/reversal
	• Monitoring of behaviour by others without feedback
	• Information about others’ approval
	• Restructuring the physical environment
*Algorithm (optional)*	• Action planning
The algorithm contained within the guidelines was reproduced with two additional features; a QR code which links directly to the guidelines and a box that contained details for local senior support. These were then laminated so that the local information could be updated as required.	• Adding objects to the environment
	• Conserving mental resources
	• Habit formation
	• Identification of self as role model
	• Prompts/cues
	• Salience of consequences
*Posters (optional)*	• Action planning
Posters with key symptoms were designed and graphics covered paediatric, adults and geriatrics. Hospitals can request the number of these posters along with the display locations. Posters also contained a QR code which linked directly to the guidelines.	• Adding objects to the environment
	• Conserving mental resources
	• Habit formation
	• Identification of self as role model
	• Prompts/cues
	• Salience of consequences
*Stickers (optional)*	• Anticipated regret
Small stickers with ‘Think brain infection’ were produced for application to blood sample bottles. These could be applied to any sample bottle as required by the hospital.	• Associative learning
	• Conserving mental resources
	• Habit formation/reversal
	• Prompts/triggers/cues
*Invitation letter (optional)*	• Action planning
A template invitation letter from the consultant inviting the junior doctor to attend each of the education session was developed for local modification. Details of the lumbar puncture box and ClickClinica were also included.	• Behavioral contract
	• Credible source
	• Goal setting
	• Information about emotional consequences
	• Information about outcomes
	• Personalised message

## Discussion

The gap between the recommended and actual management of suspected encephalitis is related to delays or diversions at susceptible junctures in the patient pathway, especially initial clinical assessment and investigation. The non-specific clinical features of suspected encephalitis and its relative rarity mean that health care professionals may fail to include it as a potential diagnosis, especially within the context of busy emergency departments. Once the diagnostic possibility is triggered, doctors can be uncertain when it is safe to perform diagnostic lumbar punctures, lack confidence in performing the procedure and experience difficulties in assembling the required kit and tests. We found fewer difficulties around starting treatment but concerns with subsequent failures to confirm diagnoses and judging when to stop treatment.

Many studies have described various barriers to and enablers of recommended practice [35], including an emerging body of work using the Theoretical Domains Framework [19-25]. Relatively fewer have demonstrated the systematic matching of identified barriers to behaviour change strategies, especially within generalisable frameworks [36-38]. As with other theoretical models and frameworks, the Theoretical Domains Framework offers a common language with which to compare findings across different study settings and targeted clinical problems. This framework has been used previously to elicit barriers and enablers within health care in a variety of settings, often for common conditions or problems, e.g. head injuries and hand hygiene [21-23,25,26,39,40]. In contrast to much earlier work, we identified memory, attention and decision processes as a key determinant of clinical behaviour when managing encephalitis; this is likely to be due to the relative rarity of the condition. The only other similar finding within the literature was within an emergency setting where a process evaluation of an unsuccessful strategy to implement the Canadian Computed Tomography Head Rule retrospectively identified this domain as a key barrier [41]. Like others studying clinical decision-making, we found that the broad domain of environmental resources and context accounted for the time pressures and distractions faced in emergency settings [21,26]. Whilst determinants of practice will inevitably vary between settings and targeted behaviours, future syntheses of studies using the framework (or other frameworks and theories) can help build a cumulative understanding of clinical behaviour [42].

Our study has six main limitations. First, our study took place in six purposively sampled UK hospitals. Factors influencing practice are likely to vary in other health care systems. However, many of the individual-level barriers we identified (e.g. around recognition and knowing when to perform lumbar punctures) are likely to be apply elsewhere.

Second, interviews elicit reported barriers and enablers; there is often a gap between perceived and actual practice [43]. For example, whilst interviewees seldom reported difficulties with initiating treatment for suspected encephalitis, recent audits indicate important delays in starting aciclovir [1,2,14,15]. We also used field observations to identify undisclosed issues. For example, we noted a gap between junior doctors’ confidence in performing a lumbar puncture and observed performance during a training session. Third, there was an additional risk of social desirability bias, whereby interviewees may have provided answers that they thought we wanted to hear, especially in relation to adherence to clinical guidance. We identified this as a particular risk with our initial focus group work, during which we suspected that interviewees responded to perceived pressure to answer correctly rather than admit to difficulties. We, therefore, conducted interviews on a one-to-one basis in a private room whenever possible. We framed questions in a non-judgmental manner and emphasised that interviews were not a test of knowledge. Fourth, our interview schedule, based upon the Theoretical Domains Framework, may not have elicited all barriers actually present within the patient pathway, especially wider, structural influences on practice. We aimed to mitigate this by taking field notes and explicitly looking for barriers across different organizational levels [27]. Overall, despite our initial concerns about the ability of the framework to capture all factors, we did not encounter any instances where any key influences on practice did not fit within the framework [44,45]. Early experience suggests that the framework can be helpful in eliciting influences on practice beyond grounded approaches [46]. However, we recognise the need for further comparative studies of intervention tailoring methods [44]. Fifth, only two researchers matched the domains to behaviour change techniques; we used a relatively informal iterative series of exchanges but recognise that a more systematic process would have resulted in a more transparent linkage between domains and behaviour change technique selection. Given that the Theoretical Domains Framework is intended to be comprehensive in describing all domains relevant to behaviour change; it initially appears odd that we only fitted around half of the behaviour change techniques to our intervention package. However, our matching process took place within a specific implementation context and thereby reflects this. Furthermore, our own cognitive biases and limitations will have affected our allocations. Sixth, in relation to our intervention building, there is no empirical work which demonstrates behaviour change techniques linked to theoretical domains are more likely to be effective than other approaches to changing clinical behaviour.

Our work went beyond the majority of published Theoretical Domains Framework work in not only eliciting barriers and enablers, but also in demonstrating further steps in intervention building. Slater and colleagues have previously illustrated a similar theoretically-guided approach to improving patient safety [35]. There is a growing assumption that tailored interventions addressing specific barriers to guideline implementation will be more effective than a non-tailored interventions [44]. A systematic review indicates that tailored interventions are more likely to improve practice compared to dissemination of guidelines as usual [47]. However, there have been no head-to-head comparisons of tailored versus non-tailored interventions. Furthermore, it is still uncertain as to whether ‘diagnostic’ work informing the development of tailored strategies actually results in implementation strategies which are both different from those developed without such an explicit process.

Sustaining interventions both during a trial and beyond is challenging [48], especially given the range of individual and organisational resources usually involved in maintaining improvement [49,50]. We therefore attempted to design an intervention package of core features which could feasibly be accommodated within a national clinical network accompanied by optional features which could be locally led and adapted to fit in with available skills and resources. We are presently undertaking a cluster randomized trial to evaluate the cost-effectiveness of this intervention package, compared to passive guideline dissemination. To assess the current management of patients with suspected encephalitis, a cluster randomised trial is currently in progress within 24 UK hospitals.

## Conclusion

Within the patient pathway, there were two critical points where practice could be improved for patients with suspected encephalitis. The first critical point was around the clinical suspicion of the condition which was influenced by the clinical environment and the limitations of professionals’ awareness and decision-making processes. The second was around the initial investigation of a lumbar puncture where medical staff often lack confidence in their abilities to perform the procedure and encounter difficulties in arranging these within busy clinical contexts. We have demonstrated the development of a tailored intervention package which specifically targets these problems and are now evaluating its cost-effectiveness.

## Additional files

Additional file 1:
**Topic guide mapped to the Theoretical Domains Framework for use during the health care professional interviews.**
Additional file 2:
**Behaviour change techniques mapped to the Theoretical Domains** **[** 28 **]** **.**
Additional file 3:
**TIDieR Template for Intervention Description and Replication** **[** 34 **]** **.**
