# Supplementary material for: The development of an intervention to promote adherence to national guidelines for suspected viral encephalitis
Source: Implement Sci. 2015 Mar 20;10:37. doi: 10.1186/s13012-015-0224-2 (PMC4373454; doi:10.1186/s13012-015-0224-2)
Supplement: Additional file 1: — Topic guide mapped to the Theoretical Domains Framework for use during the health care professional interviews. [file 13012_2015_224_MOESM1_ESM.docx]

Additional file 1: Topic guide mapped to the Theoretical Domains Framework for use during the health care professional interviews

| Cane et al., 2012 [20] | Domains | Prompts around management of suspected encephalitis patients | Prompts around  The diagnosis of suspected encephalitis: |
| --- | --- | --- | --- |
| 1 | Knowledge | You mentioned previously that you were aware of guidelines for the care of suspected encephalitis patients. Are you aware of any key messages around treatment options? (OR if no previous awareness of the guidelines – are you aware of the treatment options for suspected encephalitis patients?) | Are you aware of any guidelines around the clinical care of suspected encephalitis? If so, are you aware of any key messages around diagnosis? |
| 2 | Skills | How easy do you find it do you find it to act on the recommendation that aciclovir needs to be prescribed in six hours? | Do you know how to make a diagnosis of encephalitis? |
| 3 | Social/professional role and identity | Focusing on the prescribing and implementation of treatment, to what extent is doing this part of your usual professional role? | Focusing on the lumbar puncture, to what extent is doing a lumbar puncture part of your usual professional role? |
| 4 | Beliefs about capabilities | What would help you to remember the guidelines for encephalitis? | How confident are you that you can do a lumbar puncture within six hours of a patient being admitted? (Or help with one if asking non-medical staff) |
| 5 | Optimism | Do you anticipate that you may be able to prescribe and implement treatment for these patients into your clinical routine? | Do you anticipate/feel optimistic that you can incorporate performing lumbar punctures into your clinical routine? |
| 6 | Beliefs about consequences | How do you think you will feel if you didn’t manage to implement appropriate treatment within six hours? | What do you think about the pros and cons of doing an LP for suspected encephalitis?  (Prompt – re themselves, patients, colleagues, the organisation, positive and  negative, short and long term consequences) |
| 7 | Reinforcement | Do you feel valued if you are able to demonstrate correct management of these patients? | Do you feel valued if you perform a lumbar puncture? |
| 8 | Intentions | Are you planning to look for encephalitis when a patient presents with indicative symptoms? | Are you planning to perform more lumbar punctures? (Or, if the interviewee has answered that they feel confident and enjoy performing *lumbar punctures* previously: Are you planning on helping other members of your team to perform more *lumbar puncture*s?) |
| 9 | Goals | Do you think that you will set yourself any goals following this interview? | Have you set yourself any types of goals for performing lumbar punctures? |
| 10 | Memory, attention and decision processes | The guidelines recommend that aciclovir is prescribed within six hours (unless clinically contraindicated); is that something that you usually do? | Is following these recommendations something you usually do? |
| 11 | Environmental context and resources | Do you have the necessary resources available to you so that aciclovir can be prescribed and administered within the recommended six hour timeframe? | Are there competing demands on your time or other workplace factors that might interfere with your intention to do a lumbar puncture? |
| 12 | Social influences | Do people you work with either routinely consult the latest guidelines themselves, or encourage you to check the latest guidelines during practice? | Do people you work with encourage you to perform lumbar punctures to help diagnose or rule out encephalitis? |
| 13 | Emotion | How do you feel when you correctly diagnose or suspect encephalitis from the presenting features? | We’re interested in what health professionals feel as well as think when it comes to following best practice recommendations. So, how do you feel when you do or are planning to do a lumbar puncture?  (Further prompts: any anxieties when doing a *lumbar puncture*? (Any satisfaction or sense of achievement?) |
| 14 | Behavioral regulation | What sorts of things do you need to do before aciclovir can be prescribed? | What sorts of things do you need to do before a lumbar puncture? |
