# Supplementary material for: The development of an intervention to promote adherence to national guidelines for suspected viral encephalitis
Source: Implement Sci. 2015 Mar 20;10:37. doi: 10.1186/s13012-015-0224-2 (PMC4373454; doi:10.1186/s13012-015-0224-2)
Supplement: Additional file 2: — Behaviour change techniques mapped to the Theoretical Domains [ 28 ] . [file 13012_2015_224_MOESM2_ESM.docx]

**Additional file 2: Behaviour change techniques mapped to the Theoretical Domains [28]**

| **Identified barrier / enabler** | **Potential ‘best fit’ behaviour change techniques** |
| --- | --- |
| Knowledge | - Persuasive communication - Instruction on how to perform the behaviour - Information about antecedents |
| Skills | - Goal setting (for behaviour and outcomes) - Reward (outcome) - Graded task - Problem solving - Action planning - Behavioral contract - Feedback on behaviour - Self-monitoring of behaviour and outcomes - Instruction on how to perform a behaviour - Demonstration of the behaviour - Social comparison - Behavioral practice/ rehearsal |
| Social/professional role and identity | - Social support (unspecified) - Action planning - Persuasive communication - Personalised message - Feedback on behaviour and outcomes - Behavioural contract - Social comparison - Behaviour substitution - Credible source - Social reward - Identification of self as role model - Valued self-identity - Identity associated with changed behaviour |
| Beliefs about capabilities | - Reward (outcome) - Graded tasks - Problem solving - Social support (unspecified) - Discrepancy between current behaviour and goal - Self-monitoring of behaviour - Feedback on outcomes of behaviour - Social support (unspecified) - Social support (emotional) - behavioral experiments - Monitoring of emotional consequences - Social comparison - Exposure - Behavioral practice/rehearsal - Graded task - Reduce negative emotions - Identification of self as a role model - Framing, reframing - Incompatible beliefs - Valued self-identity - Verbal persuasion about capability - Mental rehearsal of successful performance - Focus on past success - Self-talk |
| Optimism* | - Goal setting (outcome) - Action planning - Review behaviour goal - Review outcome goal - Commitment - Feedback on behaviour - Self-monitoring of outcome of behaviour - Feedback on outcome of behaviour - Exposure - Pros and cons - Identification of self as role model - Identity associated with changed behaviour - Verbal persuasion about capability - Mental rehearsal of successful performance - Focus on past success - Self-talk |
| Beliefs about consequences | - Persuasive communication - Review behaviour goal - Discrepancy between current behaviour and goal - Review outcome goal - Feedback on behaviour - Self-monitoring of outcomes of behaviour - Feedback on outcomes of behaviour - Behavioural experiments - Information about health consequences - Salience of consequences - Information about social and environmental consequences - Anticipated regret - Information about emotional consequences - Credible source - Pros and cons - Comparative imagining of future outcomes - Reduce negative emotions - Framing/reframing - Incompatible beliefs - Vicarious consequences |
| Reinforcement* | - Review behaviour goals - Commitment - Monitoring of behaviour by others without feedback - Feedback on outcome of behaviour - Information about others’ approval - Prompts/cues - Cue signaling reward - Reduce prompts/cues - Remove access to the reward - Remove aversive stimulus - Satiation - Exposure - Associative learning - Overcorrection - Generalisation of a target behaviour - Material incentive (behaviour) - Material reward (behaviour) - Non-specific reward - Social reward - Social incentive - Non-specific incentive - Self-incentive - Incentive (outcome) - Self-reward - Reward (outcome) - Future punishment - Paradoxical instructions - Avoidance/reducing exposure to cues for the behaviour - Distraction - Behaviour cost - Punishment - Remove reward - Reward approximation - Rewarding completion - Situation specific reward - Reward incompatible behaviour - Reward alternative behaviour - Reduce reward frequency - Remove punishment - Imaginary punishment - Imaginary rearward - Vicarious consequences |
| Intentions | - Graded task - Problem solving - Social support (unspecified) - Persuasive communication - Salience of consequences - Goal setting (behaviour) - Problem solving - Goal setting (outcome) - Action planning - Behavioural contract - Commitment - Prompts/cues - Cue signaling reward - Reduce prompts/cues - Remove access to the reward - Remove aversive stimulus - Exposure - Habit formation - Habit reversal - Generalisation of a target behaviour - Graded tasks - Material incentive (behaviour) - Material reward (behaviour) - Non-specific reward - Social reward - Social incentive - Non-specific incentive - Self-incentive - Incentive (outcome) - Self-reward - Reward (outcome) - Future punishment - Avoidance/reducing exposure to cues for the behaviour - Distraction - Behaviour cost - Punishment - Remove reward - Reward approximation - Rewarding completion - Situation specific reward - Reward incompatible behaviour - Reward alternative behaviour - Reduce reward frequency - Remove punishment - Mental rehearsal of successful performance - Focus on past success - Self-talk - Imaginary punishment - Imaginary reward - Vicarious consequences |
| Goals | - Goal setting (behaviour and outcome) - Action planning - Review behaviour goals - Discrepancy between current behaviour and goal - Review outcome goal - Behavioural contract - Commitment - Self-monitoring of behaviour - Self-monitoring of outcome of behaviour - Feedback on outcomes of behaviour - Social comparison - Prompts and cues - Behavioural practice/rehearsal - Habit formation - Generalisation of a target behaviour - Graded tasks - Comparative imagining of future outcomes - Material incentive (behaviour) - Material reward (behaviour) - Non-specific reward - Social reward - Social incentive - Non-specific incentive - Self-incentive - Incentive (outcome) - Self-reward - Reward (outcome) - Future punishment - Rewarding completion - Situation specific reward - Reward incompatible behaviour |
| Memory, attention and decision processes | - Self-monitoring of behaviour and outcomes - Prompts/cues - Feedback on behavior and outcomes - Problem solving - Action planning - Commitment - Information about antecedents - Salience on consequences - Social comparison - Information about others’ approval - Prompts/cues - Exposure - Associate learning - Behavioural practice/rehearsal - Habit formation - Habit reversal - Overcorrection - Generalisation of a target behaviour - Graded tasks - Credible source - Pros and cons - Comparative imagining of future outcomes - Material incentive (behaviour) - Material reward (behaviour) - Non-specific reward - Social reward - Social incentive - Non-specific incentive - Self-incentive - Incentive (outcome) - Self-reward - Reward (outcome) - Future punishment - Reduce negative emotions - Conserving mental resources - Adding objects to the environment - Rewarding completion - Situation specific reward |
| Environmental context and resources | - Social support (unspecified) - Social support (practical) - Social support (emotional) - Social comparison - Information about others’ approval - Prompts/cues - Cue signaling reward - Conserving mental resources - Restructuring the physical environment - Restructuring the social environment - Adding objects to the environment |
| Social influences | - Reward (outcome) - Review behaviour goals - Behavioural contract - Commitment - Monitoring of behaviour by others without feedback - Feedback on behaviour - Self-monitoring of behaviour - Self-monitoring of outcomes of behaviour - Monitoring outcomes of behaviour by others without feedback - Feedback on outcomes of behaviour - Social support (unspecified) - Social support (practical) - Social support (emotional) - Social comparison - Information about others’ approval - Credible source - Social reward - Social incentive - Non-specific incentive |
| Emotion | - Commitment - Social support (emotional) - Monitoring of emotional consequences - Anticipated regret - Information about emotional consequences - Reduce negative emotions |
| Behavioural regulation | - Behavioral contract - Prompts/cues - Goal setting (outcome) - Action planning - Review behaviour goal - Discrepancy between current behaviour and goal - Review outcome goals - Commitment - Self-monitoring of behaviour - Self-monitoring of outcomes of behaviour - Feedback on outcomes of behaviour - Information about antecedents - Re-attribution - Monitoring of emotional consequences - Habit formation - Habit reversal - Overcorrection - Comparative imaging of future outcomes |

*Domain not present within Michie et al. 2008 [19], from Cane et al. 2012 [20].
