# Supplementary material for: The development of an intervention to promote adherence to national guidelines for suspected viral encephalitis
Source: Implement Sci. 2015 Mar 20;10:37. doi: 10.1186/s13012-015-0224-2 (PMC4373454; doi:10.1186/s13012-015-0224-2)
Supplement: Additional file 3: — TIDieR Template for Intervention Description and Replication [ 34 ] . [file 13012_2015_224_MOESM3_ESM.docx]

**Additional file 3: TIDieR Template for Intervention Description and Replication [34]**

|  |  | Where located? | |
| --- | --- | --- | --- |
|  |  | **Primary paper (page or appendix number)** | **Other** |
| 1 | **BRIEF NAME**  Provide the name or a phrase that describes the intervention | Page 6 |  |
| 2 | **WHY**  Describe any rationale, theory or goal of the elements essential to the intervention. | Pages 11-12 |  |
| 3 | **WHAT**  Materials: Describe any physical or informational materials used in the intervention, including those provided to participants or used in intervention delivery or in training of intervention providers. Provide I formation on where the materials can be accessed (e.g. online appendix, URL). | Table 3 |  |
| 4 | Procedures: Describe each of the procedures, activities, and/or processes used in the intervention, including any enabling or support activities. | Table 3 |  |
| 5 | **WHO PROVIDED**  For each category of intervention provider (e.g. psychologist, using assistant), describe their expertise, background and any specific training given. |  | The research team delivered the training day prior to local clinicians delivering tailored intervention components within each of their departments. |
| 6 | **HOW**  Describe the modes of delivery (e.g. face to face, or by some other mechanism, such as internet or telephone) of the intervention and whether it was provided individually or in a group. |  | Within each hospital, education was delivered by a member of the clinical team. LP boxes and posters were placed in key clinical areas and replenished by a member of the local investigators team. |
| 7 | **WHERE**  Describe the type(s) of location(s) where the intervention occurred, including any necessary infrastructure or relevant features. |  | The intervention was run in a number of departments according to hospital uptake. Consultants from at least one of the following departments led the intervention: Paediatrics; Neurology; Infectious Disease; Microbiology; Emergency Medicine; Medical Assessment Unit. |
| 8 | **WHEN and HOW MUCH**  Describe the number of times the intervention was delivered and over what period of time including the number of sessions, their schedule, and their duration, intensity or dose. |  | The education has been designed to be delivered between twice and four times a year to cover junior doctors’ rotation pattern whilst giving local flexibility. Sites can contact the research team for extra materials at any time and an update/promotional pack will be sent after six months. |
| 9 | **TAILORING**  If the intervention was planned to be personalised, titrated or adapted, then describe what, why, when and how. |  | The educational materials can be modified, however a colour coded slide set allows the integrity of the integrated behaviour change techniques to remain intact. The package has a number of core and additional components so that the package can be tailored to the site. |
| 10 | **MODIFICATIONS**  If the intervention was modified during the course of the study, describe the changes (what, why, when and how.) |  | To be detailed in the paper reporting the outcomes of the cluster randomised trial. |
| 11 | **HOW** **WELL**  Planned: If intervention adherence or fidelity was assessed, describe how and by whom, and if any strategies were used to maintain or improve fidelity, describe them. |  | To be detailed in the paper reporting the outcomes of the cluster randomised trial. |
| 12 | Actual: If intervention adherence or fidelity was assessed, describe the extent to which the intervention was delivered as planned. |  | To be detailed in the paper reporting the outcomes of the cluster randomised trial. |
|  |  |  |  |
